# Simulation modelling to identify optimal monitoring strategies: the use of the elf biomarker in liver disease monitoring

**DOI:** 10.1186/1745-6215-14-S1-O106

**Published:** 2013-11-29

**Authors:** Alice Sitch, Jac Dinnes, Julie Parkes, Walter Gregory, Jenny Hewison, Doug Altman, Jon Deeks

**Affiliations:** 1 University of Birmingham, Birmingham, UK; 2 University of Leeds, Leeds, UK; 3 University of Oxford, Oxford, UK; 4 University of Southampton, Southampton, UK

## Background

Monitoring tests are used to identify disease recurrence or progression. Monitoring strategies are complex interventions, involving specification of a test, a schedule, and a decision rule based on test results and subsequent diagnostic or therapeutic action. A systematic approach to developing a monitoring strategy is lacking however, with monitoring intervals being adopted into practice based on standard follow up schedules often with little consensus of evidence for the thresholds used to initiate treatment (Dinnes 2012).

## Aim

To identify optimal monitoring strategies for use of the Enhanced Liver Fibrosis (ELF) biomarker to monitor progression of severe liver disease for early detection of cirrhosis, which could be evaluated in an RCT.

## Method

A simulation model was constructed using evidence from the literature, existing data sources and expert opinion. Simulated data evaluated the value of sequential tests, the impact of measurement error and other sources of variability. The test schedule and decision rule were varied to identify optimal strategies. Sensitivity analyses were performed to investigate the impact of data used to influence the model and all assumptions made.

## Results

The method will be explained and results of various monitoring strategies given, identifying candidate strategies. Variability in the measurement error estimates and assumptions made regarding disease progression rates affect strategy performance. The results of sensitivity analyses will be provided.

## Conclusion

Evaluation of monitoring strategies in this way allows the combined value of all elements of a monitoring strategy to be evaluated prior to full scale investigation.

